# Toxicological Assessment of* Pseudospondias microcarpa* (A. Rich.) Engl. Hydroethanolic Leaf Extract in Rats: Haematological, Biochemical, and Histopathological Studies

**DOI:** 10.1155/2018/4256782

**Published:** 2018-05-20

**Authors:** Donatus Wewura Adongo, Priscilla Kolibea Mante, Kennedy Kwami Edem Kukuia, Charles Kwaku Benneh, Robert Peter Biney, Eric Boakye-Gyasi, Nicholas Akinwale Titiloye, Eric Woode

**Affiliations:** ^1^Department of Pharmacology, School of Medicine, University of Health and Allied Sciences, Ho, Ghana; ^2^Department of Pharmacology, Faculty of Pharmacy and Pharmaceutical Sciences, College of Health Sciences, Kwame Nkrumah University of Science and Technology, Kumasi, Ghana; ^3^Department of Pharmacology and Toxicology, University of Ghana School of Pharmacy, College of Health Sciences, University of Ghana, Accra, Ghana; ^4^Department of Pharmacology and Toxicology, School of Pharmacy, University of Health and Allied Sciences, Ho, Ghana; ^5^Department of Pharmacology, School of Medical Sciences, University of Cape Coast, Cape Coast, Ghana; ^6^Department of Pathology, School of Medical Sciences, College of Health Sciences, Kwame Nkrumah University of Science and Technology, Kumasi, Ghana

## Abstract

*Pseudospondias microcarpa* is used traditionally for treating various diseases. However, although parts of the plant are extensively used in African traditional medicine, no scientific study has been reported on its toxicity. Therefore, this study evaluated the acute and subacute toxicity studies of the ethanolic extract of* P. microcarpa* in rats. Male Sprague-Dawley rats (120–150 g) were treated orally with the extract (30, 100, 300, 1000, and 3000 mg kg^−1^) or distilled water (10 ml kg^−1^) for 2 weeks and observed daily for general appearance and signs of toxicity. In addition, blood was collected for both biochemical and haematological assays. Sections of tissues from liver, kidney, spleen, brain, and stomach were also used for histopathological examination. Administration of the extract for 14 consecutive days caused no deaths, with an LD_50_ above 3000 mg kg^−1^. Except for lymphocytes (%) that showed a significant decrease (*F*_5,23_ = 3.93, *P* = 0.013), all other haematological parameters remained unaffected by the extract. The extract at 100 mg kg^−1^ showed a significant decrease in the levels of triglyceride and very-low-density lipoproteins (both at *P* < 0.05). Weight change as well as histological evaluation of the organs indicated no toxicity. The study demonstrates that an ethanolic extract of* P*.* microcarpa* given orally to rats is safe.

## 1. Introduction

Over the last few years, there has been a tremendous rise in the acceptance and public interest in medicinal plants in both developing and developed countries. It is estimated that about 75% of the world population, primarily those of developing countries, rely on traditional remedies (mainly herbs) for the healthcare of their people [1]. In these developing countries, this has been attributed to easy access to herbal therapies, as well as cultural and economic factors. Also, in developed countries, herbal medicines are viewed as natural, time-tested, and therefore safe compared with what are perceived as synthetic drugs [2].

Although some herbal medicines have promising potential and are widely used, many of them remain untested and their use is also not monitored. In this regard, knowledge of their potential adverse effects is limited and identification of the safest and most effective therapies is difficult [3]. Medicinal plants have been shown to be capable of producing a wide range of undesirable or adverse reactions, with some being capable of causing life-threatening conditions and even death. It is therefore important to assess the toxicity of herbal medicines.

*Pseudospondias microcarpa *is one of such plants used for treating various diseases including central nervous system (CNS) disorders, arthritis, rheumatism, eye problems, kidney disorders, nasopharyngeal infections, stomach complaints, malaria, and jaundice [4]. Various studies have also shown the plant to possess antioxidant [5], antimicrobial [6], anticonvulsant [7], antidepressant [8, 9], anxiolytic [10], sedative, analgesic [11] and cytotoxic and antiplasmodial effects [12]. As demonstrated by Yondo et al. [5], we have shown in a previous study that the leaves of the plant contain some phytochemical constituents which may be responsible for its biologic activity [11].

However, although various parts of* P. microcarpa* are extensively used in African traditional medicine and despite the fact that the plant has several pharmacological actions as indicated previously, no scientific study has been reported on its toxicity. The present study therefore assessed both acute and subacute toxicity of* P*.* microcarpa *in rats.

## 2. Materials and Methods

### 2.1. Collection of Plant Material and Extraction

Fresh leaves of* P*.* microcarpa* were collected from the campus of Kwame Nkrumah University of Science and Technology (KNUST), Kumasi (6°40.626′N, 1°34.041′W), and authenticated at the Department of Herbal Medicine, Faculty of Pharmacy and Pharmaceutical Sciences, College of Health Sciences, KNUST, Kumasi, Ghana. A voucher specimen (KNUST/HM1/2013/L005) was kept at the herbarium of the faculty.

Leaves of the plant were room-dried for seven days and pulverised into fine powder. The powder was extracted by cold percolation with 70% (v/v) ethanol in water over a period of 72 h and the resulting extract concentrated into a syrupy mass under reduced pressure at 60°C in a rotary evaporator. It was further dried in a hot air oven at 50°C for a week and kept in a refrigerator for use. The yield was 20.5% (w/w). In this study, the crude extract is subsequently referred to as PME or extract.

### 2.2. Animals

Male Sprague-Dawley rats (120–150 g) were purchased from the Noguchi Memorial Institute for Medical Research, Accra, Ghana, and kept in the animal house of the Department of Pharmacology, Kwame Nkrumah University of Science and Technology, Kumasi, Ghana. The animals were housed in groups of 5 in stainless steel cages (34 × 47 × 18 cm^3^) with soft wood shavings as bedding and housing conditions controlled: temperature maintained at 24-25°C, relative humidity of 60–70%, and 12 h light-dark cycle. They had free access to tap water and food (commercial pellet diet, GAFCO, Tema, Ghana). A period of at least one week for adaptation to the laboratory facilities was allowed. The research was conducted in accordance with accepted principles for laboratory animal use and care [13]. Approval for this study was obtained from the Faculty Ethics Committee.

### 2.3. Acute Toxicity Study

Sprague-Dawley rats were orally treated with the extract (30, 100, 300, 1000, and 3000 mg kg^−1^) or distilled water (10 ml kg^−1^) and placed in observation cages. The rats were evaluated for general pharmacological and physiological behaviours as well as mortality at 0, 15, 30, 60, 120, and 180 min, up to 24 h after treatment.

### 2.4. Subacute Toxicity

Sprague-Dawley rats were put into six groups of 5 animals each. Five experimental groups were given PME orally at doses of 30, 100, 300, 1000, and 3000 mg kg^−1^ for 2 weeks. The control group received distilled water orally at the volume of 10 mL kg^−1^. During the experimental period, animals were observed daily for general appearance and signs of toxicity.

#### 2.4.1. Preparation of Serum and Isolation of Organs

At the end of the study, animals were fasted overnight and sacrificed by cervical dislocation. About 1.5 mL of blood was collected into vacuum tubes containing 2.5 *μ*g of ethylenediaminetetraacetic acid (EDTA) as an anticoagulant for haematological assay and 3.5 ml into sample tubes containing a separating gel for biochemical parameters. The blood for the biochemical parameters was centrifuged (4000 rpm at 4°C for 10 min) to obtain serum and stored at −20°C.

Organs harvested included liver, kidney, brain, stomach, and spleen.

#### 2.4.2. Haematological Assay

Haematological analysis was performed with the haematological analyser, ABX micros ES 60 (HORIBA Medical Diagnostics, France). The parameters examined included white blood cells (WBC), red blood cells (RBC), haematocrit (HCT), haemoglobin (HGB), mean cell volume (MCV), mean cell haemoglobin (MCH), mean cell haemoglobin concentration (MCHC), lymphocytes (LMP), platelet distribution width (PDW), red cell (erythrocyte volume) distribution width, relative volume of thrombocytes (PCT), platelets (PLT), and mean platelet (thrombocyte) volume (MPV).

#### 2.4.3. Biochemical Assay

Biochemical values were measured with a COBAS INTEGRA 400 (Hoffmann-La Roche Ltd., Basel, Switzerland), which assessed levels of alkaline phosphatase (ALP), alanine transaminase (ALT), aspartate transferase (AST), gamma-glutamyl transferase (GGT), total protein, albumin, globulin, total bilirubin (T-Bil), direct bilirubin (D-Bil), indirect bilirubin (I-Bil), creatinine, urea, triglyceride (TG), cholesterol, high-density lipoproteins (HDL), low-density lipoproteins (LDL), and very-low-density lipoproteins (VLDL).

#### 2.4.4. Body and Organ Weight Assessment

Brain, liver, kidney, stomach, heart, and spleen were isolated and weighed. Body weights of the rats were taken on days 0 and 15. Relative organ weight (ROW) was then calculated:

(1) ROW=absolute  organ  weight  gbody  weight  of  animal  on  sacrifice  day  g×100.

#### 2.4.5. Histopathological Examinations

Sections of the tissue from liver, kidney, spleen, brain, and stomach were used for histopathological examination. Tissues were fixed in 10% neutral buffered formalin (pH 7.2) and dehydrated through a series of ethanol solutions, embedded in paraffin, and routinely processed for histological analysis. Sections of 2 *μ*m thickness were cut and stained with haematoxylin-eosin for examination. The stained tissues were observed through an Olympus microscope (BX-51) and photographed by INFINITY 4 USB Scientific Camera (Lumenera Corporation, Ottawa, Canada).

### 2.5. Statistical Analysis

In all experiments, a sample size of 5 animals was utilized. Data are presented as mean ± SEM. The presence of significant differences among means of groups was determined by one-way ANOVA using GraphPad Prism for Windows version 5 (GraphPad Software, San Diego, CA, USA). Significant difference between pairs of groups was calculated using the Newman-Keuls' multiple comparison test. *P* < 0.05 was considered statistically significant.

## 3. Results

### 3.1. Acute Toxicity

Treatment of rats with the extract produced sedation and analgesia at all doses used (Table 1). No deaths were recorded over the 24 h observation period, indicating an LD_50_ above 3000 mg kg^−1^.

### 3.2. Subacute Toxicity

No deaths were recorded after 14 days of treatment with the extract. Other signs of toxicity were absent except sedation, which was observed throughout the treatment period.

#### 3.2.1. Effect of Extract on Body Weight and Organ Weight

**Figure 1:**
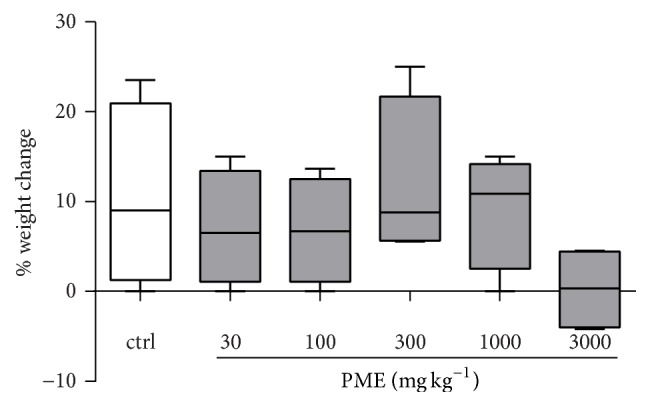
Effect of 14-day treatment with PME on the % change in body weights of rats in the subacute toxicity test. Data are expressed as mean ± SEM (*n* = 5). Treated groups were compared to controls with a one-way ANOVA followed by Newman-Keuls' test. The lower and upper margins of the boxes represent the 25th and 75th percentiles, while the extended arms represent the 10th and 90th percentiles, respectively.

As shown in Figure 1, the extract had no significant effect on body weight change, although this parameter was decreased at the highest dose (3000 mg kg^−1^). Treatment with the extract increased weight of the spleen at 100, 300, and 1000 mg kg^−1^ (all at *P* < 0.05) when compared to the control group (Table 2).

#### 3.2.2. Effect of Extract on Haematological Parameters

Except for lymphocytes (%) that showed a significant decrease (*F*_5,23_ = 3.93, *P* = 0.013), all other parameters remained unaffected by the extract (Table 3).

#### 3.2.3. Effect of Extract on Biochemical Parameters

Alanine transaminase (ALT) and aspartate transferase (AST) levels were decreased but the decrease was not statistically significant when compared to the control group (Table 4). Bilirubin levels at the doses of 30 and 100 mg kg^−1^ were also decreased, although ANOVA showed no significant difference. ANOVA showed a significant decrease in the levels of triglyceride (*F*_5,23_ = 3.086, *P* = 0.034) and VLDL (*F*_5,23_ = 3.834, *P* = 0.015) with Newman-Keuls' post hoc analysis, revealing significance at 100 mg kg^−1^ (both at *P* < 0.05).

#### 3.2.4. Histopathological Changes

**Figure 2:**
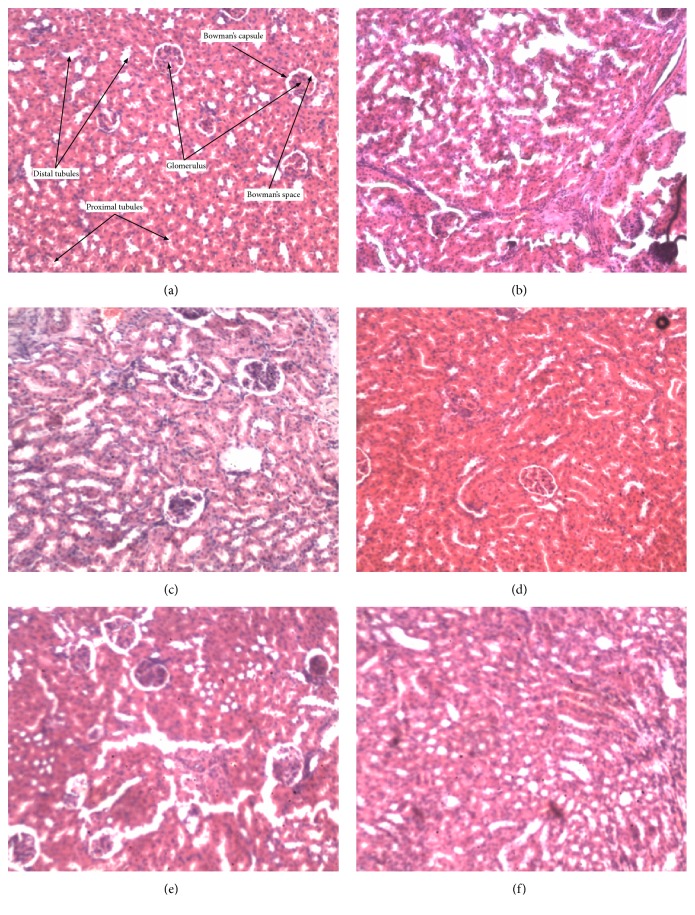
Photomicrograph of the sections of the kidney in control rats (a) and rats treated orally with 30 mg kg^−1^ (b), 100 mg kg^−1^ (c), 300 mg kg^−1^ (d), 1000 mg kg^−1^ (e), and 3000 mg kg^−1^ (f) of the extract for 14 days in the subacute toxicity study (H&E, ×400).

**Figure 3:**
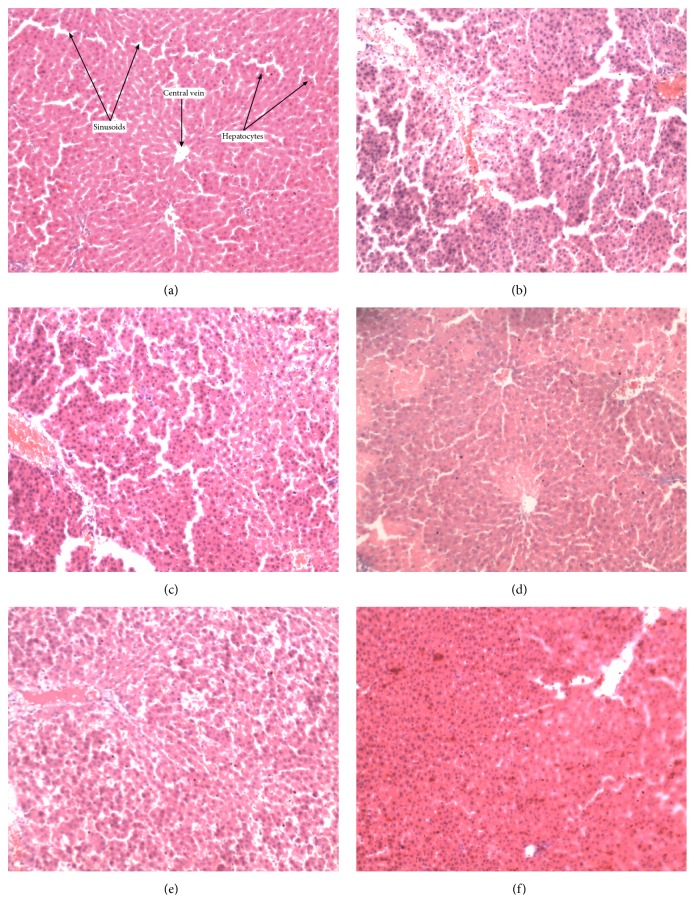
Photomicrograph of the sections of the liver in control rats (a) and rats treated orally with 30 mg kg^−1^ (b), 100 mg kg^−1^ (c), 300 mg kg^−1^ (d), 1000 mg kg^−1^ (e), and 3000 mg kg^−1^ (f) of the extract for 14 days in the subacute toxicity study (H&E, ×400).

**Figure 4:**
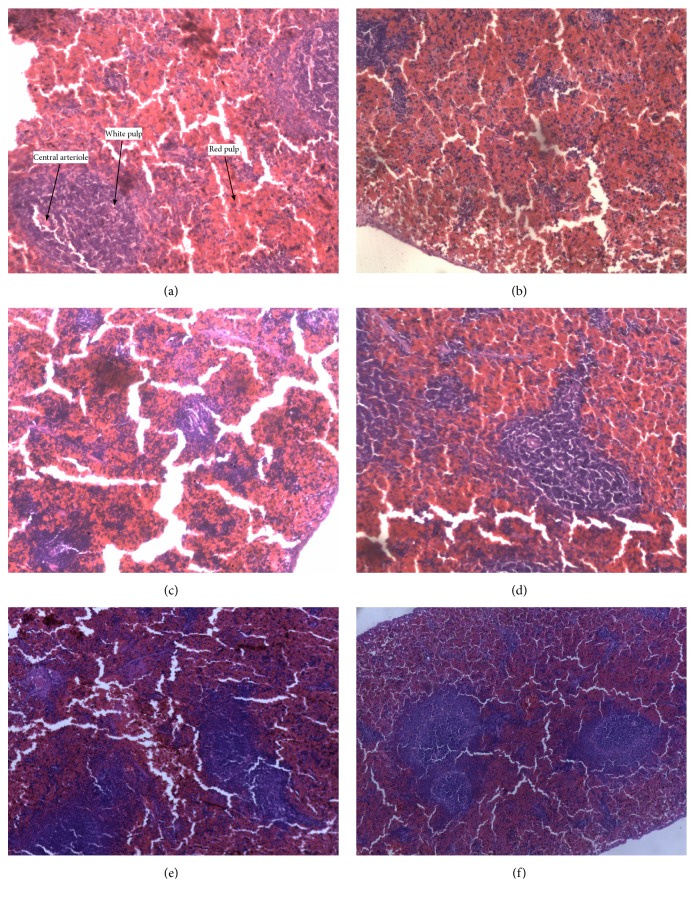
Photomicrograph of the sections of the spleen in control rats (a) and rats treated orally with 30 mg kg^−1^ (b), 100 mg kg^−1^ (c), 300 mg kg^−1^ (d), 1000 mg kg^−1^ (e), and 3000 mg kg^−1^ (f) of the extract for 14 days in the subacute toxicity study (H&E, ×400).

**Figure 5:**
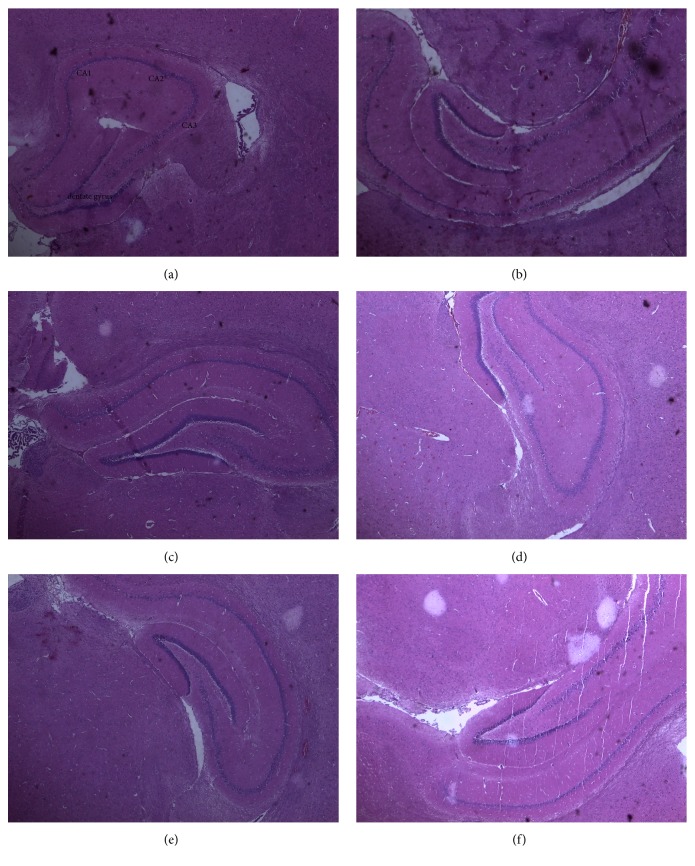
Photomicrograph of the sections of the brain in control rats (a) and rats treated orally with 30 mg kg^−1^ (b), 100 mg kg^−1^ (c), 300 mg kg^−1^ (d), 1000 mg kg^−1^ (e), and 3000 mg kg^−1^ (f) of the extract for 14 days in the subacute toxicity study (H&E, ×400).

**Figure 6:**
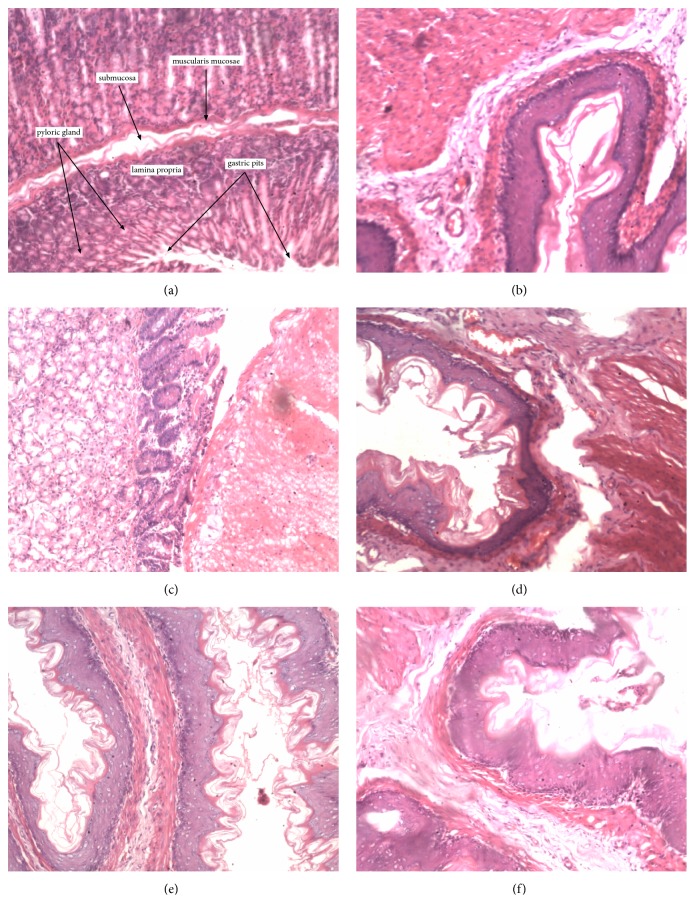
Photomicrograph of the sections of the stomach in control rats (a) and rats treated orally with 30 mg kg^−1^ (b), 100 mg kg^−1^ (c), 300 mg kg^−1^ (d), 1000 mg kg^−1^ (e), and 3000 mg kg^−1^ (f) of the extract for 14 days in the subacute toxicity study (H&E, ×400).

Figures 2–6 show the photomicrographs of sections of the isolated organs of control and PME-treated rats for the 14-day subacute toxicity study. Histopathological evaluation of the organs isolated from rats sacrificed at the end of the subacute toxicity study revealed no significant extract-related morphological changes compared to the control animals.

Sections from the splenic tissue (Figure 4) were essentially normal, showing preservation of the lymphoid follicles. Few follicular enlargements as well as minimal dilation of the sinusoids were observed. All sections of the kidneys (Figure 2) showed essentially normal glomerulus, tubules, and blood vessels. Inflammation and necrosis were also absent. Furthermore, no casts in the tubules as well as other deposits in the kidneys were observed. Brain sections showed well-preserved brain tissue (Figure 5). No significant morphological changes like necrosis and red neurons were observed in all the doses of the extract as compared to the control. With regard to the stomach (Figure 6), there was essentially no ulceration and inflammation. In addition, the mucosa glands and muscularis propria showed no abnormalities. At all the doses used, the extract showed no tendency to induce gastritis. As shown in Table 5, the specific morphological changes assessed in the liver included fatty change, hydropic swelling, inflammation, and fibrosis. The extract-induced inflammation was mainly restricted to the portal triad with occasional spill over into the hepatocytes in all the doses. Mild fibrosis (at 3000 mg kg^−1^) and mild fatty change (at 1000 and 3000 mg kg^−1^) were observed in some liver sections. In addition, hydropic swelling was observed in all rats treated with PME at 1000 and 3000 mg kg^−1^. The toxic effect of the extract on the liver was relatively mild and only trace evidence was seen at high doses (1000 and 3000 mg kg^−1^).

## 4. Discussion

Medicinal plants are often considered safe for human use due to their natural origin. However, various reports suggest the potential risks involved with such plants [14]. Thus, there is a need to assess the safety of these medicinal plants before use. This study therefore assessed both acute and subacute toxicity studies of* Pseudospondias microcarpa* hydroethanolic leaf extract (PME) in rats.

In the acute toxicity study, rats treated with PME showed signs of sedation and analgesia, suggesting possible central nervous system (CNS) depressant and analgesic effects. This confirms a previous study in our laboratory [11], as well as traditional use of the plant. The LD_50_ of the plant extract, given orally, was found to be above 3000 mg kg^−1^. At the relatively high doses used, the plant extract caused no mortality and appeared to cause no apparent toxicity, suggesting that PME is relatively nontoxic.

In toxicological evaluation, it is always important to assess subacute toxicity profile of test compounds, since repeated dosing helps to evaluate morphological and physiological changes in organs [15]. Therefore, a subacute toxicity study was performed in rats and, similar to observations in the acute toxicity test, treatment with the extract for 14 days caused no mortality. In addition, water and food consumption was normal.

Body weight change of animals was also assessed, since changes in the body weights can be used as an indicator of adverse or toxic effects of drugs [16, 17]. In this study, it was observed that the change in body weight of animals treated with the extract was not significant when compared to the control group, indicating absence of any severe adverse effects. Aside the body weight change, the liver, spleen, kidney, brain, and stomach are the primary organs affected by metabolic reactions induced by toxicants. Therefore, the relative organ weight is an important index of physiological and pathological status in man and animals [18–20]. In this study, PME treatment caused no significant difference in organ-to-body ratio of the various organs when compared to the control group, except for the spleen, which showed an increase. However, gross pathological examination of the spleen of rats treated with the extract did not reveal any abnormalities, presence of lesions, or changes in colour.

Analysis of blood parameters in animal studies is important to evaluate the risk of alterations of the haematological system in human toxicity and also to explain blood related functions of a plant extract or its isolates [21–23]. Thus, various haematological indices were measured. No significant difference was found in the majority of haematological parameters between treated and control groups except for the observed decrease in lymphocytes (%). Lymphocytes are the main effector cells of the immune system and thus protect the body from infections [24]. Decreased lymphocytes in the present study may therefore suggest a low infection resistance, since the effector cells of the immune system might be affected. Haematological parameters such as MCHC, MCH, and MCV relate to individual red blood cells, whereas HGB, RBC, PCV, and RCDW are linked to the total population of red blood cells. Thus, the unchanged effect of the extract on these parameters may imply that neither the incorporation of haemoglobin into red blood cells nor the morphology and osmotic fragility of the red blood cells were altered [25, 26]. This therefore excludes the possibility of anaemia or disturbance linked to erythrocytes and indicates nontoxic effects of PME on the haematopoietic system.

Liver and kidney functions tests as well as serum lipid profile are important parameters in determining the safety of plant extracts or their isolates [27, 28]. To evaluate kidney function, measurements of urea and creatinine levels in the blood are usually performed [29]. These two parameters are usually increased to four or five times the normal values in control animals in cases of acute or chronic renal toxicity [17]. This study shows that treatment with* Pseudospondias microcarpa* extract in rats for 14 days does not produce possible kidney malfunction, since the biomarkers of kidney function (urea and creatinine) were not affected.

The activities of AST and ALT are the most sensitive tests employed in the diagnosis of hepatic diseases [30]. With damaged liver cell plasma, various enzymes normally located in the cytosol are released into the blood, resulting in increased enzyme levels in the serum [31, 32]. Thus, increased levels of ALT, AST, ALP, and GGT may be interpreted as a result of liver cell destruction or changes in the membrane permeability [30, 33]. Measurement of these enzymes in the serum is therefore a useful quantitative marker of the extent and type of hepatocellular damage. Treatment with the extract had no effect on the levels of these serum enzymes, indicating nontoxic effects on liver function.

An important physiologic role of the liver is the removal of toxic endogenous and exogenous substances from the blood. Thus, tests based on excretory functions of the liver are related to bilirubin metabolism [34]. Bilirubin is the product of haem following the breakdown of red blood cells by phagocytic cells. It is carried by serum albumin to the liver, where most of it is conjugated with glucuronide prior to excretion into the bile. Increased levels in the blood result in jaundice and could be due to increased haemolysis of red blood cells, primary hepatocellular damage, or mechanical biliary duct obstruction [35]. Therefore, this metabolite serves as a good indicator to assess the functional capacity of the liver. In this study, serum levels of direct, indirect, and total bilirubin after treatment with the extract for 14 days were not elevated, indicating that PME did not have any deleterious effects on hepatic metabolism or biliary excretion. However, although not significantly, the extract decreased levels of direct, indirect, and total bilirubin, especially at the low doses used (30 and 100 mg kg^−1^). The bilirubin-lowering effect as well as decreased ALT levels could suggest possible hepatoprotective effects of the extract, confirming traditional use of the plant for treating jaundice [4].

Cholesterol, apart from acting as a precursor for the synthesis of bile acids, hormones, and vitamins, is also an essential component of most biological membranes [36, 37]. However, an increase in its levels could lead to atherosclerosis, hyperproteinaemia, cirrhosis, haemolytic jaundice, malnutrition, and increased risk of cardiovascular diseases [38, 39]. As the primary carrier of plasma cholesterol, low-density lipoprotein (LDL) is often referred to as bad cholesterol, since increased levels cause atherosclerosis [40]. In addition, elevated LDL levels have been reported to be associated with hepatic lesion and damage [31, 41]. Furthermore, increased levels of serum triglyceride lead to hyperlipidaemia and low levels imply that there is no risk factor related to atherosclerosis [38]. In the present study, decreased levels of triglycerides, LDL, and VLDL particularly at the low dose of 100 mg kg^−1^ coupled with normal cholesterol levels further confirm normal liver function and decreased risk of atherosclerosis. The decreased levels of VLDL and LDL cholesterols could also indicate hypolipidemic effects of the extract.

Evaluation of pathological alterations induced in laboratory animals by novel drugs represents the basis of their safety assessment before they can be used in the clinical setting and this is largely based on conventional histopathological techniques [42, 43]. Therefore, histological analysis was done to further examine the pathological state of the various organs. Except for the liver that showed mild fatty change, hydropic swelling, and mild inflammation at the highest doses (1000 and 3000 mg kg^−1^), detailed gross and histopathological examination of the various organs showed no significant pathological changes. These changes in liver are mild and may not be considered clinically significant, since serum levels of hepatic enzymes (ALT and AST), which are considered markers of liver function, were not significantly elevated. However, caution should be taken in using this extract beyond 3000 mg kg^−1^. In addition, chronic toxicity studies should be done in rats and other species to ascertain the safety of the plant extract.

## 5. Conclusion

Results of this study show that oral administration of the hydroethanolic leaf extract of* Pseudospondias microcarpa* is relatively safe in rats. However, caution should be exercised when extrapolating this result to man.

## Figures and Tables

**Table 1:** Effects of *Pseudospondias microcarpa* hydroethanolic leaf extract in the primary observation test in rats.

Dose (mg kg^−1^)	Mortality D/T	Effects
0	0/5	No change
30	0/5	Sedation, analgesia
100	0/5	Sedation, analgesia
300	0/5	Sedation, analgesia
1000	0/5	Sedation, analgesia
3000	0/5	Sedation, analgesia

D/T: dead/treated.

**Table 2:** Effects of PME on relative organ weights (ROW) of rats in the subacute toxicity test.

Organs	Control	PME (mg kg^−1^)				
		30	100	300	1000	3000
Liver	3.03 ± 0.26	2.66 ± 0.11	2.90 ± 0.09	3.05 ± 0.18	3.05 ± 0.10	2.75 ± 0.05
Kidney	0.65 ± 0.01	0.62 ± 0.02	0.68 ± 0.01	0.72 ± 0.02	0.70 ± 0.02	0.66 ± 0.02
Spleen	0.48 ± 0.04	0.44 ± 0.02	0.65 ± 0.02^*∗*^	0.69 ± 0.09^*∗*^	0.69 ± 0.02^*∗*^	0.45 ± 0.03
Stomach	1.08 ± 0.05	1.04 ± 0.04	1.13 ± 0.08	1.17 ± 0.07	1.13 ± 0.04	1.07 ± 0.05
Brain	0.93 ± 0.03	0.87 ± 0.02	0.94 ± 0.03	1.01 ± 0.06	1.00 ± 0.06	0.88 ± 0.05

Data are presented as mean ± SEM (*n* = 5). ^*∗*^*P* < 0.05 is considered statistically significant from control.

**Table 3:** Effect of 14-day treatment with PME on haematological parameters in rats.

Parameters	Control	PME (mg kg^−1^)				
		30	100	300	1000	3000
WBC (10^3^/mm^3^)	4.90 ± 1.66	4.63 ± 1.18	5.10 ± 1.32	4.68 ± 0.36	5.40 ± 1.19	6.40 ± 1.28
LYM (%)	90.33 ± 4.02	86.85 ± 1.12	77.0 ± 2.66^*∗*^	73.3 ± 2.07^*∗*^	75.7 ± 2.07^*∗*^	74.8 ± 3.14^*∗*^
RBC (10^6^/mm^3^)	7.49 ± 1.07	7.21 ± 0.21	6.41 ± 0.19	6.33 ± 0.46	6.54 ± 0.39	7.28 ± 0.23
HGB (g/dL)	13.08 ± 1.57	12.68 ± 0.33	11.43 ± 0.19	11.20 ± 0.88	12.00 ± 0.90	12.58 ± 0.55
HCT (%)	43.73 ± 5.27	41.83 ± 2.19	37.40 ± 1.43	37.78 ± 2.48	38.43 ± 0.66	40.25 ± 1.67
MCV (*µ*m^3^)	59.00 ± 2.04	57.75 ± 1.65	58.25 ± 0.94	60.25 ± 2.06	59.25 ± 3.04	55.25 ± 1.03
MCH (pg)	17.58 ± 0.51	17.58 ± 0.41	17.85 ± 0.29	17.68 ± 0.18	18.25 ± 0.48	17.28 ± 0.44
MCHC (g/dL)	30.33 ± 0.60	30.45 ± 0.91	30.70 ± 0.86	29.60 ± 0.99	31.10 ± 1.98	31.20 ± 0.89
RDW (%)	16.03 ± 0.87	16.25 ± 0.87	16.70 ± 0.68	16.68 ± 0.57	15.93 ± 0.45	13.80 ± 1.15
PLT (10^3^/mm^3^)	883.3 ± 52.71	963.0 ± 165.7	800.0 ± 127.3	848.5 ± 103.8	875.5 ± 139.5	850.8 ± 89.91
MPV (*µ*m^3^)	6.80 ± 0.30	7.43 ± 0.27	6.78 ± 0.39	6.73 ± 0.32	6.95 ± 0.33	7.58 ± 0.25
PCT (%)	0.60 ± 0.04	0.71 ± 0.12	0.54 ± 0.07	0.58 ± 0.09	0.62 ± 0.11	0.64 ± 0.06
PDW (%)	10.85 ± 0.66	10.85 ± 1.77	10.93 ± 0.96	11.63 ± 0.52	10.30 ± 2.52	9.15 ± 2.30

Data are presented as mean ± SEM (*n* = 5). ^*∗*^*P* < 0.05 is considered statistically significant from control.

**Table 4:** Effect of 14-day treatment with PME on biochemical parameters in rats.

Parameters	Control	PME (mg kg^−1^)				
		30	100	300	1000	3000
AST (U/L)	244.9 ± 43.44	273.30 ± 9.17	191.70 ± 11.1	250.0 ± 23.06	196.40 ± 8.28	200.90 ± 3.82
ALT (U/L)	122.4 ± 30.54	103.1 ± 17.78	77.70 ± 7.96	106.6 ± 10.85	86.15 ± 6.86	95.0 ± 23.82
ALP (U/L)	171.6 ± 28.38	207.3 ± 38.76	176.4 ± 14.01	207.3 ± 31.67	197.3 ± 35.09	168.7 ± 22.60
GGT (U/L)	0.97 ± 0.33	0.78 ± 0.24	1.45 ± 0.47	1.20 ± 0.33	0.95 ± 0.22	1.43 ± 0.43
Total pr. (g/L)	66.98 ± 0.47	67.23 ± 2.63	61.63 ± 2.60	63.08 ± 1.78	62.80 ± 2.25	64.18 ± 1.46
Albumin (g/L)	43.83 ± 1.11	45.33 ± 2.20	41.83 ± 1.73	41.95 ± 1.73	39.88 ± 2.13	38.30 ± 1.13
Globulin (g/L)	23.10 ± 1.14	21.73 ± 1.51	19.80 ± 0.91	21.15 ± 0.79	22.93 ± 3.81	25.88 ± 2.11
Bil. (*µ*mol/L)	1.00 ± 0.39	0.62 ± 0.12	0.48 ± 0.16	1.13 ± 0.44	0.88 ± 0.34	0.78 ± 0.26
D-Bil (*µ*mol/L)	0.33 ± 0.13	0.22 ± 0.05	0.18 ± 0.05	0.45 ± 0.24	0.28 ± 0.11	0.20 ± 0.07
I-Bil (*µ*mol/L)	0.67 ± 0.26	0.40 ± 0.08	0.30 ± 0.11	0.68 ± 0.34	0.60 ± 0.23	0.58 ± 0.19
Urea (mmol/L)	6.85 ± 0.94	7.33 ± 0.47	7.61 ± 0.67	7.74 ± 0.40	8.40 ± 0.87	8.54 ± 0.43
Creat. (mmol/L)	47.25 ± 6.20	48.75 ± 6.32	46.25 ± 5.43	53.75 ± 7.56	41.75 ± 5.89	51.25 ± 5.39
Chol. (mmol/L)	2.20 ± 0.14	2.36 ± 0.16	1.97 ± 0.13	2.20 ± 0.28	2.20 ± 0.17	1.89 ± 0.07
TG (mmol/L)	0.60 ± 0.03	0.57 ± 0.05	0.4 ± 0.01^*∗*^	0.56 ± 0.04	0.61 ± 0.06	0.53 ± 0.02
HDL (mmol/L)	1.70 ± 0.02	1.86 ± 0.10	1.62 ± 0.10	1.56 ± 0.10	1.64 ± 0.13	1.57 ± 0.07
LDL (mmol/L)	0.20 ± 0.10	0.23 ± 0.06	0.18 ± 0.04	0.39 ± 0.02	0.29 ± 0.08	0.25 ± 0.15
VLDL (mmol/L)	0.20 ± 0.01	0.26 ± 0.02	0.1 ± 0.01^*∗*^	0.24 ± 0.02	0.27 ± 0.02	0.23 ± 0.01
Coronary risk	1.30 ± 0.07	1.25 ± 0.05	1.22 ± 0.02	1.42 ± 0.12	1.35 ± 0.06	1.17 ± 0.02

Data are presented as mean ± SEM (*n* = 5). ^*∗*^*P* < 0.05 is considered statistically significant from control.

**Table 5:** Histopathological results of the liver in rats orally treated with PME for 2 weeks.

Dose (mg kg^−1^)	Fatty change	Hydropic swelling	Mild inflammation	Mild fibrosis
Saline	0/4	0/4	0/4	0/4
30	0/4	0/4	0/4	0/4
100	0/4	0/4	4/4	0/4
300	0/4	1/4	4/4	0/4
1000	2/4	4/4	4/4	0/4
3000	2/4	4/4	4/4	2/4

Results are expressed as the number of rats with pathological findings per total number of rats sectioned.

## References

[1] Gilani A. H., Rahman A. U. (2005). Trends in ethnopharmacology. *Journal of Ethnopharmacology*.

[2] Schachter S. C. (2009). Botanicals and herbs: a traditional approach to treating epilepsy. *Neurotherapeutics*.

[3] WHO. Traditional Medicine Strategy 2002-2005. WHO/EDM/TRM/20021. 2002

[4] Gyllenhaal C., Burkill H. M. (1999). The Useful Plants of West Tropical Africa Volume 2 Families E - I. *Kew Bulletin*.

[5] Yondo J., Fomekong G. I. D., Kontangui M-C., Wabo J. P., Tankoua O. F., Kulate J-R. (2009). *In vitro antioxidant potential and phytochemical constituents of three cameroonian medicinal plants used to manage parasitic diseases. Annals of Nutrition and Metabolism*.

[6] Kisangau D. P., Hosea K. M., Joseph C. C., Lyaruu H. V. M. (2007). *In vitro* antimicrobial assay of plants used in traditional medicine in Bukoba rural district, Tanzania. *African Journal of Traditional, Complementary and Alternative Medicines*.

[7] Adongo D. W., Mante P. K., Kukuia K. K. E. (2017). Anticonvulsant activity of Pseudospondias microcarpa (A. Rich) Engl. hydroethanolic leaf extract in mice: The role of excitatory/inhibitory neurotransmission and nitric oxide pathway. *Journal of Ethnopharmacology*.

[8] Adongo D. W., Kukuia K. K. E., Mante P. K., Ameyaw E. O., Woode E. (2015). Antidepressant-Like Effect of the Leaves of Pseudospondias microcarpa in Mice: Evidence for the Involvement of the Serotoninergic System, NMDA Receptor Complex, and Nitric Oxide Pathway. *BioMed Research International*.

[9] Adongo D. W., Mante P. K., Kukuia K. K. E., Woode E. Preclinical evidence of a rapid-onset antidepressant-like effect of Pseudospondias microcarpa hydroethanolic leaf extract in a chronic depression model.

[10] Adongo D. W., Mante P. K., Kukuia K. K. E., Ameyaw E. O., Woode E., Azi I. H. (2016). Anxiolytic-like effect of the leaves of Pseudospondias microcarpa (A. Rich.) Engl. in mice. *Journal of Basic and Clinical Physiology and Pharmacology*.

[11] Adongo D. W., Mante P. K., Woode E., Ameyaw E. O., Kukuia K. K. E. (2014). Effects of hyrdroethanolic leaf extract of Pseudospondias microcarpa (A. Rich.) Engl. (Anacardiaceae) on the central nervous system in mice. *The Journal of Phytopharmacology*.

[12] Malebo H. M., Tanja W., Cal M. (2009). Antiplasmodial, anti-trypanosomal, anti-leishmanial and cytotoxicity activity of selected Tanzanian medicinal plants.. *Tanzania Journal of Health Research*.

[13] NRC (2010). *Guide for the Care and Use of Laboratory Animals*.

[14] Jordan S. A., Cunningham D. G., Marles R. J. (2010). Assessment of herbal medicinal products: challenges, and opportunities to increase the knowledge base for safety assessment. *Toxicology and Applied Pharmacology*.

[15] Kouadio J. H., Bleyere M. N., Kone M., Dano S. D. (2014). Acute and sub-acute toxicity of aqueous extract of Nauclea latifolia in Swiss mice and in OFA rats. *Tropical Journal of Pharmaceutical Research*.

[16] Adedapo A. A., Abatan M. O., Idowu S. O., Olorunsogo O. O. (2005). Toxic effects of chromatographic fractions of Phyllanthus amarus on the serum biochemistry of rats. *Phytotherapy Research*.

[17] Arsad S. S. (2014). Histopathologic Changes in Liver and Kidney Tissues from Male Sprague Dawley Rats Treated with Rhaphidophora Decursiva (Roxb.) Schott Extract. *Journal of Cytology & Histology*.

[18] Dybing E., Doe J., Groten J. (2002). Hazard characterisation of chemicals in food and diet: dose response, mechanisms and extrapolation issues. *Food and Chemical Toxicology*.

[19] Jothy S. L., Zakaria Z., Chen Y., Lau Y. L., Latha L. Y., Sasidharan S. (2011). Acute oral toxicity of methanolic seed extract of Cassia fistula in mice. *Molecules*.

[20] Konaté K., Bassolé I. H. N., Hilou A. (2012). Toxicity assessment and analgesic activity investigation of aqueous acetone extracts of Sida acuta Burn f . and Sida cordifolia L. (Malvaceae), medicinal plants of Burkina Faso. *BMC Complementary and Alternative Medicine*.

[21] Olson H., Betton G., Robinson D. (2000). Concordance of the toxicity of pharmaceuticals in humans and in animals. *Regulatory Toxicology and Pharmacology*.

[22] Afzan A., Abdullah N. R., Halim S. Z. (2012). Repeated dose 28-days oral toxicity study of *Carica papaya* L. leaf extract in Sprague Dawley rats. *Molecules*.

[23] Yakubu M. T., Afolayan A. J. (2009). Effect of aqueous extract of bulbine natalensis baker stem on haematological and serum lipid profile of male Wistar rats. *Indian Journal of Experimental Biology (IJEB)*.

[24] Hayes A. W., Kruger C. L. (2014). *Hayes' Principles and Methods of Toxicology*.

[25] Adebayo J. O., Adesokan A. A., Olatunji L. A., Buoro D. O., Soladoye A. O. (2005). Effect of ethanolic extract of *Bougainvillea spectabilis* leaves on haematological and serum lipid variables in ratsitm. *Biokemistri*.

[26] Ashafa A. O. T., Sunmonu T. O., Afolayan A. J. (2011). Effects of leaf and berry extracts of phytolacca dioica l. on haematological and weight parameters of wistar rats. *African Journal of Pharmacy and Pharmacology*.

[27] Patel C., Dadhaniya P., Hingorani L., Soni M. G. (2008). Safety assessment of pomegranate fruit extract: acute and subchronic toxicity studies. *Food and Chemical Toxicology*.

[28] Syahida M., Maskat M. Y., Suri R., Mamot S., Hadijah H. (2012). Soursop (Anona muricata L.): Blood hematology and serum biochemistry of sprague-dawley rats. *International Food Research Journal*.

[29] Emeigh Hart S. G. (2005). Assessment of renal injury in vivo. *Journal of Pharmacological and Toxicological Methods*.

[30] Kim N.-Y., Lee M.-K., Park M.-J. (2005). Momordin Ic and oleanolic acid from Kochiae Fructus reduce carbon tetrachloride-induced hepatotoxicity in rats. *Journal of Medicinal Food*.

[31] Brinda R., Vijayanandraj S., Uma D., Malathi D., Paranidharan V., Velazhahan R. (2013). Role of adhatoda vasica (L.) nees leaf extract in the prevention of aflatoxin-induced toxicity in Wistar rats. *Journal of the Science of Food and Agriculture*.

[32] Ashok Shenoy K., Somayaji S. N., Bairy K. L. (2001). Hepatoprotective effects of Ginkgo biloba against carbon tetrachloride induced hepatic injury in rats. *Indian Journal of Pharmacology*.

[33] Lim H.-K., Kim H.-S., Choi H.-S. (2000). Effects of acetylbergenin against D-galactosamine-induced hepatotoxicity in rats. *Pharmacological Research*.

[34] Naik P. (2012). *Essentials of Biochemistry 1st Edition*.

[35] Jansen P. L., Bittar E. E., Bittar E. E. (2004). *Bilirubin metabolism: The Liver in Biology and Disease*.

[36] Repa J. J., Mangelsdorf D. J. (2000). The role of orphan nuclear receptors in the regulation of cholesterol homeostasis. *Annual Review of Cell and Developmental Biology*.

[37] Zarzecki M. S., Araujo S. M., Bortolotto V. C., de Paula M. T., Jesse C. R., Prigol M. (2014). Hypolipidemic action of chrysin on Triton WR-1339-induced hyperlipidemia in female C57BL/6 mice. *Toxicology Reports*.

[38] Sodipo O. A., Abdulrahman F. I., Sandabe U. K., Akinniyi J. A. (2011). Total lipid profile and faecal cholesterol with aqueous fruit extract of solanum macrocarpum in triton-induced hyperlipidaemic albino rats. *Journal of Medicinal Plants Research*.

[39] Woo M.-N., Bok S.-H., Choi M.-S. (2009). Hypolipidemic and body fat-lowering effects of Fatclean in rats fed a high-fat diet. *Food and Chemical Toxicology*.

[40] Toyin Y. M., Adewumi A. M., Temidayo O. A. (2008). Alterations in serum lipid profile of male rats by oral administration of aqueous extract of fadogia agrestis stem. *Research Journal of Medicinal Plant*.

[41] Edrington T. S., Kamps-Holtzapple C. A., Harvey R. B., Kubena L. F., Elissalde M. H., Rottinghaus G. E. (1995). Acute hepatic and renal toxicity in lambs dosed with fumonisin-containing culture material.. *Journal of Animal Science*.

[42] Greaves P. (2011). Histopathology of Preclinical Toxicity Studies: Interpretation and Relevance in Drug Safety Evaluation: Fourth Edition. *Histopathology of Preclinical Toxicity Studies: Interpretation and Relevance in Drug Safety Evaluation: Fourth Edition*.

[43] Prabu P. C., Panchapakesan S., Raj C. D. (2013). Acute and sub-acute oral toxicity assessment of the hydroalcoholic extract of Withania somnifera roots in wistar rats. *Phytotherapy Research*.

